# Acute kidney injury survivors should have long-term follow-up

**DOI:** 10.1186/s13054-014-0703-x

**Published:** 2014-12-19

**Authors:** Wim Vandenberghe, Eric AJ Hoste

**Affiliations:** Department of Intensive Care Medicine, Ghent University Hospital, Ghent University, 9000 Ghent, Belgium; Research Foundation-Flanders, 100 Brussels, Belgium

## Abstract

Acute kidney injury (AKI) is a frequently occurring complication in ICU patients and is associated with decreased short- and long-term survival. Gammelager and colleagues showed that AKI patients are at increased risk for developing heart failure and myocardial infarction at long-term follow-up. Their study provides strong epidemiological data on cardiorenal syndrome type 3, and their findings help explain the worse long-term survival of AKI patients. Finally, it also highlights the need for specific follow-up programs for ICU survivors.

In a recent article, Gammelager and colleagues [1] investigated the association between acute kidney injury (AKI) and long-term cardiac morbidity and stroke in a representative ICU cohort. AKI occurs in one- to two-thirds of ICU patients and is associated with worse outcome [2,3]. Short-term worse outcomes can be explained by the effects of decreased kidney function, such as volume overload and retention of uremic toxins [4]. Long-term outcomes are probably affected by development of chronic kidney disease [5]. Recently, there has been increased interest in the complex interaction between the kidney and heart. AKI leading to acute cardiac events has been termed cardiorenal syndrome type 3 (CRS-3) [6]. At present this concept is only sparsely supported by human data [7]. The study by Gammelager and colleagues is one of the first providing high-quality data on CRS-3 in ICU patients.

Several groups, including the group of Gammelager and colleagues, have demonstrated worse long-term outcomes for AKI patients [3,8-11]. The present study by Gammelager and colleagues demonstrates that cardiovascular disease may contribute to these worse outcomes. Over a 3-year period, AKI stage 1 and greater was associated with heart failure (hazard ratio 1.33), and AKI stages 2 and 3 were associated with myocardial infarction (hazard ratio 1.51). Similar findings were reported before by James and colleagues [12] in a cohort of non-ICU patients after coronary angiography. The paper by Gammelager and colleagues is one of the first providing long-term epidemiologic data on CRS-3 in ICU patients. Importantly, it shows that CRS-3 is also relevant for patients discharged from the ICU, a less well-recognized aspect of CRS-3.

These findings are strengthened by the methodological quality of the study. Selection bias was limited by including a large multicenter ICU cohort, and a population-based medical registry guaranteed virtually complete patient follow-up. Studies using different AKI definitions cannot be compared. Therefore, it is crucial that the universally accepted KDIGO (Kidney Disease: Improving Global Outcomes) definition for AKI was used [13].

A limitation is that administrative data were used for recoding of the endpoints. Administrative databases may be limited by both over- and under-reporting, and also miss detailed information on, for example, severity of heart failure. Also, an epidemiologic study can only demonstrate an association, rather than prove a causal effect, in this case between AKI and cardiac events. These data on CRS-3 are therefore hypothesis generating and should prompt further research on the pathophysiologic mechanisms explaining the worse cardiovascular outcomes.

How can we explain this increased risk for cardiovascular events? This may be mediated, especially in the long-term, by chronic kidney disease developing after AKI, but other factors may also play a role [5]. In the acute phase, AKI may exert a negative impact on the heart, leading to cellular response with apoptosis, remodeling and fibrosis, which may ultimately lead to arrhythmias, conduction abnormalities, heart failure, and ischemia [7,14].

The study by Gammelager and colleagues is also one of the few that reports on the association between AKI and stroke, but showed no association during the 3-year follow-up. These findings are in contrast to those found in Taiwan by Wu and colleagues [15], where in a matched case-controlled study AKI patients had a higher risk and higher severity of stroke than non-AKI patients. An important difference with the cohort of Gammelager and colleagues was that the study cohort included only severe AKI treated with renal replacement therapy and was not limited to ICU patients. Severity of AKI may therefore play a role in risk for stroke.

Another important lesson that can be learned from these long-term outcome data is that AKI survivors should have long-term follow-up. We were already aware that follow-up of kidney function is important, but these data also highlight the importance of cardiovascular follow-up. As other types of ICU survivors also have specific long-term morbidity issues, this highlights the need for specific and multidisciplinary follow-up programs for ICU survivors.

## Conclusion

Gammelager and colleagues showed that AKI survivors are at increased risk for myocardial infarction and heart failure in the years following ICU discharge. These findings may contribute to decreased long-term survival of AKI patients, and also highlights the need for specific follow-up programs for ICU survivors.

## Abbreviations

AKI: Acute kidney injury
CRS-3: Cardiorenal syndrome type 3
